# Effect of acute normovolemic hemodilution on anesthetic effect, plasma concentration, and recovery quality in elderly patients undergoing spinal surgery

**DOI:** 10.1186/s12877-023-04397-w

**Published:** 2023-10-24

**Authors:** Tong Liu, Yu Bai, Lei Yin, Jin-Huo Wang, Na Yao, Lai-Wei You, Jian-Rong Guo

**Affiliations:** Department of Anesthesiology and Perioperative Medicine, Shanghai Gongli Hospital, Naval Military Medical University, No.219 Miaopu Road, Pudong New Area, Shanghai, 200135 China

**Keywords:** Acute normovolemic hemodilution, Anesthetic effect, Elderly, Plasma concentration, Spinal Surgery

## Abstract

**Objective:**

To explore the effect of acute normovolemic hemodilution (ANH) on the anesthetic effect, plasma concentration, and postoperative recovery quality in elderly patients undergoing spinal surgery.

**Methods:**

A total of 60 cases of elderly patients aged 65 to 75 years who underwent elective multilevel spinal surgery were assigned randomly into the ANH group (n = 30) and control group (n = 30). Hemodynamic and blood gas analysis indexes were observed and recorded before ANH (T_1_), after ANH (T_2_), immediately after postoperative autologous blood transfusion (T_3_), 10 min (T_4_), 20 min (T_5_), 30 min (T_6_), 40 min (T_7_), and 50 min (T_8_) after the transfusion, and at the end of the transfusion (i.e., 60 min; T_9_). At T_3 ~ 9_, bispectral index (BIS) and train-of-four (TOF) stimulation were recorded and the plasma propofol/cisatracurium concentration was determined. The extubation time and recovery quality were recorded.

**Results:**

The ANH group presented a lower MAP value and a higher SVV value at T_2_, and shorter extubation and orientation recovery time (*P* < 0.05) compared with the control group. BIS values at T_8_ and T_9_ were lower in the ANH group than those in the control group (*P* < 0.05). TOF values at T_7 ~ 9_ were lower in the ANH group than those in the control group (*P* < 0.05). There were no statistically significant differences in the postoperative plasma concentrations of propofol and cisatracurium between the groups (*P* > 0.05).

**Conclusion:**

During orthopedic surgery, the plasma concentration of elderly patients is increased after autologous blood transfusion of ANH, and the depth of anesthesia and muscle relaxant effect are strengthened, thus leading to delayed recovery of respiratory function and extubation.

## Introduction

With an aging population there is an increase in the proportion of elderly patients undergoing orthopedic surgery [1, 2]. As orthopedic multilevel spinal surgery is characterized by complex dissection, abundant blood supply, and a large surgical wound, and additionally as the intraoperative bleeding is difficult to control, the surgery is often accompanied by massive intraoperative bleeding. All these affect the surgical field of vision, reduce the precision of the surgical operation, increase the requirement for blood transfusions, and lead to transfusion-related adverse reactions [3]. In addition, elderly patients are less tolerant to ischemia and hypoxia and may have some underlying diseases. Perioperative bleeding also increases the incidence of relevant postoperative symptoms—for instance, it may cause epidural hematoma, dural sac compression, neurological dysfunctions, and even pose a threat to life in severe cases. Therefore, it is critical to effectively reduce intraoperative blood loss and protect patients’ blood. Acute normovolemic hemodilution (ANH) is a good blood conservation technique that has been widely used in clinical practice [4]. ANH involves removing autologous blood from a patient after inducing anesthesia while diluting blood with an equal volume of plasma substitute-artificial colloid fluid, and reducing blood loss by decreasing the loss of active components of erythrocytes during bleeding, then returning the autologous blood containing whole blood to the patient immediately upon completion of the surgical procedures [5]. This method can improve microcirculation that can increase oxygen supply to tissues and organs, [6] reduce the need for allogeneic blood transfusion [7] thereby mitigating complications such as infection, transfusion reaction, and disease transmission caused by allogeneic blood transfusion, [8] and effectively save blood resources. It has gradually developed into a safe and effective measure for blood conservation [9].

We found in our clinical work that patients experience deeper anesthesia, and delayed muscle relaxant recovery and tracheal catheter removal after autologous blood transfusion in ANH [10]. The following reasons have been considered: the drug effect increases after hemodilution and the anesthetic in autologous blood works or the drug metabolism and efficacy change after autologous blood transfusion. There are few studies on the effect of ANH autologous blood transfusion on the depth of anesthesia and muscle relaxant effect in surgical patients. In this study, our objective was to observe the effect of postoperative autologous blood transfusion in moderate ANH on the depth of anesthesia, muscle relaxant effect, and plasma concentration in elderly patients undergoing orthopedic surgery, to provide a theoretical basis for rapid recovery of patients as well as reasonable and safe use of the ANH technique.

## Data and methods

### General data

This study was approved by the Ethics Committee of Shanghai Pudong Gongli Hospital (2018 Gongli Hospital LSZ No. glll-26). All patients signed informed consent forms. A total of 60 cases of patients who were aged 65 to 75 years, weighed 55 to 80 kg, and who underwent ASA grade I to II elective spinal surgery were selected. The estimated intraoperative blood loss was ≥ 600 ml. None of the patients had hematological system diseases or anemia (Hb ≥ 110 g/L) and had hematocrit (Hct) ≥ 35%. They had good nutritional status, normal blood routine, normal liver and kidney function, and grade 1 to 2 cardiac function on examination. None of them had myasthenia gravis, hypoproteinemia, psychiatric disorders, or other diseases.

### Grouping and treatment

The patients were randomized into two groups using the random number table: the ANH group that received moderate acute normovolemic hemodilution and the routine control group that received no hemodilution. In the ANH group, blood was removed through the radial artery from patients at a speed of 200 mL/10 min after stably inducing anesthesia. The formula of blood volume collected is as follows:

Blood volume collected = estimated blood volume (EBV) × 2 × (Hct_actual_ - Hct_set_) / (Hct_actual_ + Hct_set_). EBV is estimated blood volume in the human body, which is calculated by body weight. EBV is 70 mL/kg for males and 60 mL/kg for females.

Hct_actual_ is hematocrit measured by preoperative blood gas analysis, and Hct_set_ is hematocrit set after hemodilution. In our study, the set Hct is 28–30%. The collected blood was stored in ACD blood storage bags at room temperature. Hydroxyethyl starch 130/0.4 injection was transfused at a ratio of 1:1 (colloidal fluid to blood volume collected). Hydroxyethyl starch 130/0.4 injection or compound sodium lactate was infused at 10 ml·kg^− 1^·h^− 1^ during the operation, to maintain hemodynamic stability. Patients in the ANH group were transfused with the collected autologous blood immediately after the operation. The control group was transfused with allogeneic blood when Hct was less than 25%. The fluctuation of intraoperative blood pressure was controlled within 20% of the base value.

### Anesthesia method

All the patients were subject to fasting for 6 h and water deprivation for 2 h before surgery and did not take preoperative medication. After a patient was transferred to the operating room, the blood pressure (BP), electrocardiogram (ECG), heart rate (HR), and oxygen saturation (SpO_2_) were monitored, BIS and TOF (OrganonTeknika, the Netherlands) monitors were connected. Then, the patient underwent puncture and catheterization of right internal jugular vein under local anesthesia for monitoring CVP and transfusion, and puncture and catheterization of left radial artery and connection to FloTrac/Vigil cardiac output monitoring system. The operator opened the main interface and entered basic information of the patient and monitored invasive arterial blood pressure after zero setting. After computer-controlled breathing was achieved, the stroke volume variation (SVV) and cardiac output (CO) were monitored after zero setting. The same team of orthopedic surgeons operated on all patients. Anesthesia induction: midazolam 0.05 mg/kg, plasma target-controlled concentration of propofol 3 µg/mL (batch No.: 19,105,050; B.Baunmelsungen AG), sufentanil 0.5 µg/kg, cisatracurium 0.2 mg/kg (batch No.: 190709AK, Jiangsu Hengrui Pharmaceuticals Co., Ltd.). When the TOF value decreased to 0, tracheal intubation and mechanical ventilation were performed, V_T_ was set to 8 ~ 10 mL/kg, breathing rate to 12 ~ 15 bpm, and I: E to 1:2, ventilation parameters were adjusted and P_ET_CO_2_ was maintained at 35 ~ 45 mmHg. Anesthesia maintenance: Propofol was infused at a target plasma concentration of 2.5 µg/mL using a TCI pump. Remifentanil was continuously pumped at 0.2 µg·kg^− 1^·min^− 1^ using a microinfusion pump, and cisatracurium besylate was administered at 2 µg·kg^− 1^·min^− 1^. The BIS value was maintained between 45 ~ 60. Pumping of anesthetic drugs were stopped after the operation. Nasopharyngeal temperature was monitored and maintained at no less than 35℃. Circulatory stability was maintained during the operation. Neostigmine and atropine were not used for antagonism after spontaneous breathing resumed. The patients had cough reflex recovery, clear mind, and protective reflexes recovery, V_T_ > 5 mL/kg, and RR of 14 ~ 20 bpm. The tracheal catheter was removed.

### Monitoring indexes

Changes in average MAP, HR, SVV, and CO before ANH (T_1_), after ANH (T_2_), at autologous blood transfusion immediately after the operation (T_3_), and 30 min (T_6_) and 60 min (T_9_) after autologous blood transfusion were monitored, and their values were recorded. The autologous blood transfusion was completed 60 min after the operation. Changes in BIS and TOF values at T_3_, 10 min (T_4_), 20 min (T_5_), 30 min (T_6_), 40 min (T_7_), and 50 min (T_8_) after transfusion, and T_9_ were observed and recorded. The blood gas analysis indexes before anesthesia (T_0_) and at T_9_ were observed and recorded, and the time from the end of the operation to removal of the tracheal catheter in the two groups was recorded. The recovery of patients was assessed using the Aldrete scoring table. Liquid chromatogram tandem mass spectrometry (LC-MS/MS) method was established to investigate the method specificity, standard curve and linearity range, residues, lower limit of quantification, precision, and accuracy, and to determine the plasma concentration of propofol and cisatracurium at T_3_ ~ T_9_ after the operation.

### Statistical treatment

Data processing was performed using SPSS 26.0 statistical software. The measured data in the normal distribution were expressed as mean ± standard deviation ($\stackrel{-}{x}\pm s$). T-test was used for comparison between the two groups, and analysis of variance was adopted for comparison between multiple groups. The enumeration data were expressed with the number of cases, and ${x}^{2}$ test was used for comparison between groups. *P*<0.05 was interpreted as a statistically significant difference.

## Results

### Comparison of general data

There were no significant differences in body weight, age, gender, ASA grade, Hct, TP, albumin (ALB), and Hb between the two groups (*P* > 0.05). (Table 1)

**Table 1:** Comparison of general conditions (n = 30, $\stackrel{-}{x}\pm s$)

Groups	Weight (kg)	Age (year)	Gender (male/female)	ASA grade (I/ II)	Hct(%)	TP (g/L)	ALB (g/L)	Hb (g/L)
ANH group	62.36 ± 7.72	64.4 ± 7.2	12/18	17/13	42.54 ± 3.41	65.79 ± 5.00	39.89 ± 2.52	139.57 ± 11.97
Control group	61.93 ± 8.15	66.3 ± 6.5	14/16	14/16	39.95 ± 3.54	64.81 ± 3.97	38.61 ± 2.57	137.00 ± 11.22

### Comparison of intraoperative fluid infusion and urine volume between the two groups

There was no statistically significant difference in postoperative urine volume between the ANH group and the control group (*P* > 0.05). There was no statistically significant difference in postoperative blood volume in the suction bottle between the two groups (*P* > 0.05). The total fluid infusion volume in the ANH group significantly increased compared with that of the control group (*P* < 0.05). (Table 2)

**Table 2:** Comparison of intraoperative fluid volume between the two groups (n = 30, $\stackrel{-}{x}\pm s$)

Volume	ANH group	Control group	*P*
Urine(ml)	711 ± 92	736 ± 80	0.448
Bleeding(ml)	404 ± 94	443 ± 119	0.330
Total infusion(ml)	2514 ± 339	1980 ± 361*	0.000
Autogenous blood collection(ml)	950.00 ± 159.32		

^*^P < 0.05, Compared with Control group

### Hemodynamic changes of the two groups at the time points

At T_2_, MAP (mmHg) in the ANH group significantly decreased when compared with the control group, that is, the MAP (mmHg) after blood collection in the acute normovolemic hemodilution group was lower than that in the control group; the difference was statistically significant (*P* < 0.05). There were no statistically significant differences in MAP (mmHg) at the other time points between the ANH group and control group (*P* > 0.05). SVV (%) at T_2_, T_3_, T_6_, and T_9_ in the ANH group was significantly higher than that in the control group (*P* < 0.05). There was no statistically significant difference in the change in SVV% at T_1_ between the two groups (*P* > 0.05). There were no statistically significant differences in HR (bpm) and CO (L/min) between the ANH group and control group (*P* > 0.05). (Table 3)

**Table 3:** Comparison of hemodynamic index between the two groups (n = 30, $\stackrel{-}{x}\pm s$)

Item	Groups	T_1_	T_2_	T_3_	T_6_	T_9_
MAP(mmHg)	ANH group	87 ± 10	76 ± 9	84 ± 12	114 ± 20^abc^	109 ± 17^abc^
	Control group	84 ± 5	90 ± 10*	91 ± 15	122 ± 10^abc^	114 ± 12^abc^
HR(bpm)	ANH group	69 ± 12	66 ± 9	63 ± 10	75 ± 21	79 ± 14^b^
	Control group	63 ± 7	60 ± 7	59 ± 13	72 ± 15^bc^	77 ± 13^abc^
CO(L/min)	ANH group	4.5 ± 1.1	4.2 ± 1.4	5.0 ± 2.0	6.9 ± 2.2^abc^	6.7 ± 1.9^abc^
	Control group	4.4 ± 1.8	4.6 ± 1.9	4.9 ± 2.3	6.0 ± 2.1^a^	6.3 ± 2.0^ab^
SVV(%)	ANH group	8.7 ± 1.6	11.7 ± 2.2	11.6 ± 4.5	7.9 ± 5.1^b^	8.7 ± 3.1
	Control group	7.9 ± 2.7	6.7 ± 2.7*	7.5 ± 1.7*	3.9 ± 1.0*^abc^	6.0 ± 2.0*^a^

Compared with ANH group, *P < 0.05; Compared with T1, ^a^*P* < 0.05; Compared with T_2_, ^b^*P* < 0.05; Compared with T_3_, ^c^*P* < 0.05

### Comparison of anesthesia depth (BIS) and muscle relaxant effect (TOF) between the two groups

**Fig. 1:**
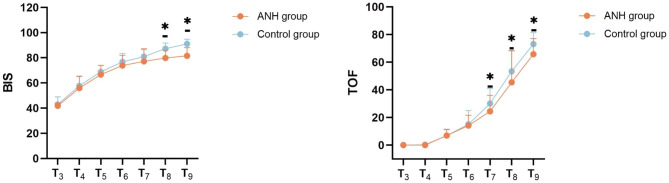
Comparison of BIS and TOF values between the two groups at different time points. ^*^P < 0.05, Compared with ANH group

The BIS values at T_8_ and T_9_ in the ANH group were significantly less than those in the control group (*P* < 0.05). There were no statistically significant differences in BIS values at T_3_ ~ T_7_ between the ANH group and the control group (*P* > 0.05). The TOF values at T_7_ ~ T_9_ in the ANH group were significantly less than those in the control group (*P* < 0.05). There were no statistically significant differences in TOF values between the ANH group and the control group at T_4_ ~ T_6_ (*P* > 0.05). (Fig. 1)

### Method validation and test results of propofol and cisatracurium plasma concentration determination

**Fig. 2:**
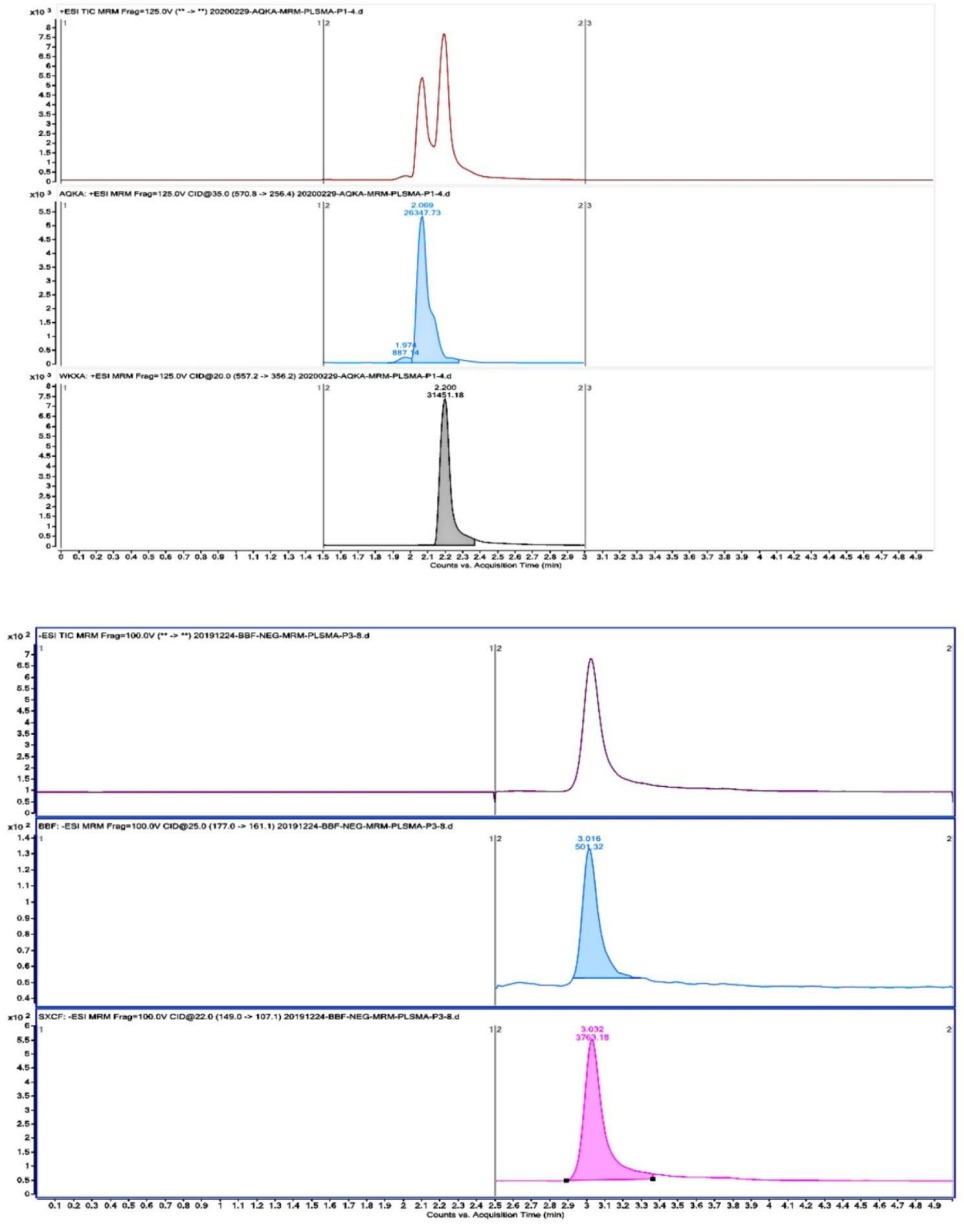
Left:Typical chromatograms of propofol in human plasma (top: total ion flow, Middle: propofol channel, bottom: thymol channel); Right:Typical chromatogram of Cisatracurium besylate in human plasma (top: total ion flow, Middle: Cisatracurium besylate channel, bottom: Vecuronium channel)

The method for determining the concentration of propofol and cisatracurium in plasma was highly specific and showed good linearity (100 ~ 10000ng/ml) without obvious residual effect, while the lower limit of quantification and the deviation of precision and accuracy were within 15%. Therefore, the method met the sample analysis requirements. (Fig. 2)

### Comparison of postoperative propofol and cisatracurium concentration between the two groups

**Fig. 3:**
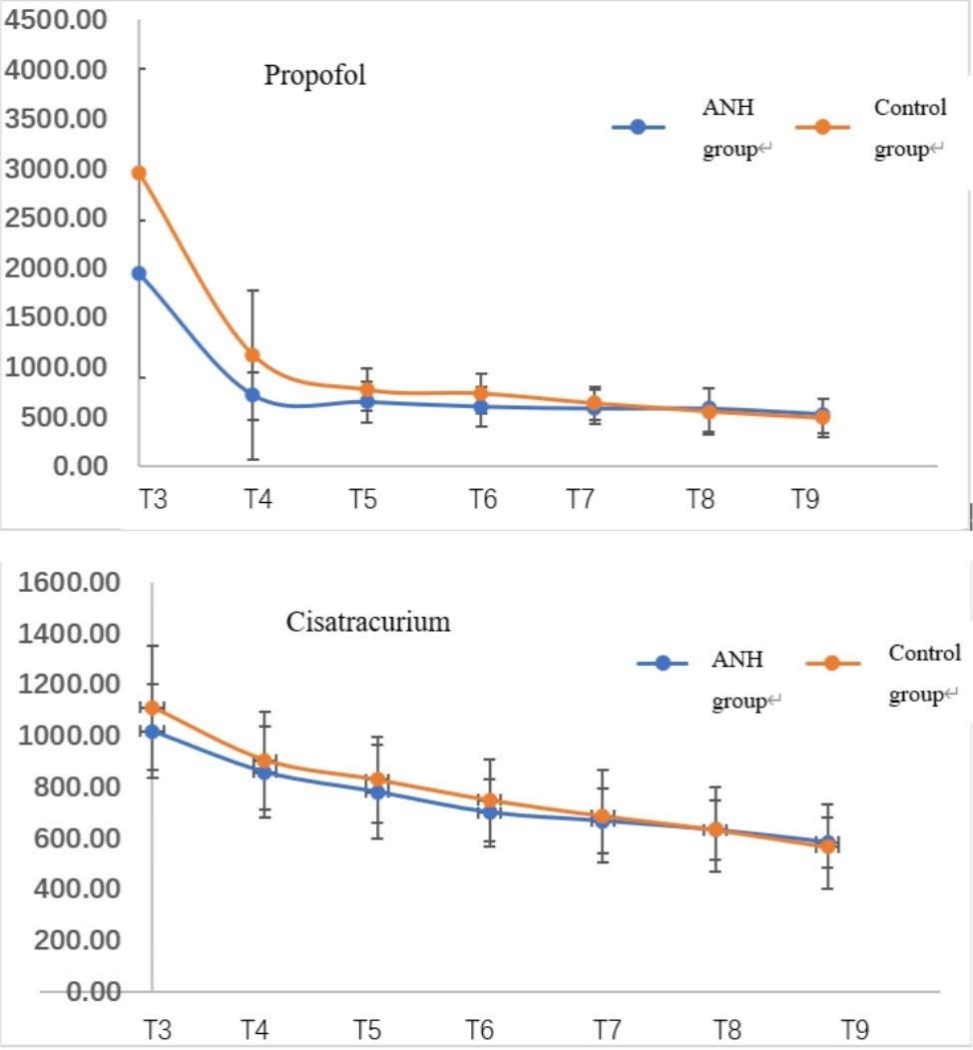
Comparison of blood concentrations of propofol and cisatracurium between the two groups at different time points (n = 30, $\stackrel{-}{x}\pm s$)

The concentration of propofol at T_3_ in the ANH group was significantly lower than that in the control group (*P* < 0.05). There were no statistically significant differences in the concentration of propofol at T_4_ ~ T_9_ between the two groups (*P* > 0.05). There were no statistically significant differences in the concentration of cisatracurium at T_3_ ~ T_9_ between the ANH group and control group (*P* > 0.05). (Fig. 3)

### Comparison of blood gas analysis indexes between the two groups

There were no statistically significant differences in PaO_2_ (mmHg) at T_9_ and T_0_ between the two groups (*P* > 0.05). There was no statistically significant difference in PaO_2_ (mmHg) at T_9_ between the ANH group and the control group (*P* > 0.05). The differences in PaCO_2_ (mmHg) and Lac (mmol/L) values at T_9_ and T_0_ between the two groups were statistically significant (*P* < 0.05). The Lac (mmol/L) value at T_9_ in the ANH group was significantly higher than that in the control group (*P* < 0.05). The pH values at T_9_ in the two groups were significantly reduced when compared with T_0_ (*P* < 0.05). (Table 4)

**Table 4:** Comparison of PaO_2_, PaCO_2_, pH and lactic acid value between the two groups (n = 30, $\stackrel{-}{x}\pm s$)

Item	Groups	T_0_	T_9_
PaO_2_(mmHg)	ANH group	74.06 ± 7.86	72.28 ± 12.06
	Control group	75.79 ± 8.58	73.83 ± 9.79
PaCO_2_(mmHg)	ANH group	38.48 ± 4.85	42.71 ± 4.18*
	Control group	38.85 ± 3.28	43.34 ± 3.65*
pH	ANH group	7.425 ± 0.34	7.349 ± 0.42*
	Control group	7.421 ± 0.34	7.361 ± 0.30*
Lac(mmol/L)	ANH group	0.96 ± 0.31	2.92 ± 1.38*
	Control group	0.99 ± 0.45	1.78 ± 1.04^a^*

Compared with ANH group, ^a^*P* < 0.05; Compared with T_0_, *P < 0.05

### Comparison of postoperative recovery degree indexes between the two groups

The time of tracheal catheter removal in the ANH group was significantly delayed compared to the control group (*P* < 0.05). The Aldrete score in the ANH group was lower than that of the control group (*P* < 0.05). (Table 5)

**Table 5:** Comparison of Extubation time and recovery score of the two groups (n = 30, $\stackrel{-}{x}\pm s$)

Groups	ANH group	Control group	*P*
Extubation time(min)	54.57 ± 10.23	35.67 ± 7.10*	0.000
Aldrete score	7.36 ± 1.01	8.87 ± 0.83*	0.000

Compared with ANH group, *P < 0.05

## Discussion

Spinal surgery, characterized by a large wound, long operative time, abundant blood supply in bone tissue, and difficulty in stopping bleeding, is often accompanied by massive blood loss [11]. Allogeneic blood transfusion is often required to offset blood loss. In recent years, the increasing clinical demand for blood has caused a shortage of banked blood, and allogeneic blood transfusion may lead to adverse reactions such as infection, transfusion reaction, and disease transmission [12]. For these reasons, the use of autologous blood is the preferred method in clinical surgeries [13]. ANH is a common method of autologous blood transfusion in surgery. As it can obviously reduce allogeneic blood transfusions and is characterized by ease of operation, low economic cost, and highly cost-effective clinical use, ANH has been widely used in spinal surgery among the elderly.

Hemodynamics is an important monitoring index during anesthesia, and can reflect the circulation status of patients. Stable hemodynamics is not only the prerequisite for effective perfusion of vital organs and tissues, but also closely related to the success of surgery and postoperative recovery. At present, FloTrac/Vigileo cardiac output monitoring system has been widely adopted in thoracic surgery, heart surgery, sepsis, and other fields, as it is easy to operate, requires no manual correction, and has a good correlation with pulmonary artery catheterization in patients with normal cardiac function [14]. Studies have shown that SVV is significantly correlated with blood volume, and can accurately reflect the volume status of individuals and responsiveness to fluid therapy [15]. In spinal surgery, SVV can accurately predict fluid responsiveness during prone position surgery [16, 17]. In this study, although an equal volume of colloidal solution was supplemented after ANH, the ANH group presented a significant decrease in MAP immediately after hemodilution and an increase in SVV%. Changes in these indexes suggest insufficient volume. The main reason for the above hemodynamic changes was that the patients were under anesthesia and the compensatory role of the body did not come into play. Anesthetics such as sufentanil and propofol have a direct inhibitory effect on the myocardium, and the sympathetic nerve is also inhibited. In addition to these factors, dilation of capacity vessels after anesthesia, decreased cardiac compensatory function of elderly patients, reduced vessel capacity caused by blood collection, and blood collection speed, contribute to insufficient blood volume even after an equal volume of colloid fluid is infused after ANH. The comparison of data on intraoperative fluid infusion and urine volume of the two groups can also demonstrate whether elderly orthopedic patients should be given more fluid supplementation to maintain stable hemodynamics after ANH. Therefore, special attention should be paid to hemodynamic changes during blood collection and after hemodilution.

The appropriate depth of anesthesia must be maintained when the patient is under general anesthesia during surgery. The depth of anesthesia mainly reflects the inhibitory degree of sedatives on cerebral cortex function [18]. Both, too deep and too shallow anesthesia can have adverse effects. Too deep anesthesia may lead to depression of respiratory circulation, delayed recovery, neurological injury, and postoperative delirium. On the other hand, too shallow anesthesia may lead to intraoperative awareness, psychological disorders, drastic fluctuations of circulatory system, and release of abundant inflammatory factors [19]. BIS can use EEG signals representing different sedation levels subject to digital processing to deduce the depth of anesthesia [20]. It is an internationally recognized indicator for monitoring the depth of anesthesia [21]. The BIS value between 40 and 60 is generally considered to be the clinically appropriate depth of anesthesia [22]. Studies have revealed that there is a correlation between the depth of anesthesia and the plasma concentration of anesthetics. Currently, the depth of anesthesia maintained by propofol, the most widely used intravenous anesthetic in clinical practice, is closely correlated with BIS [23]. Dahaba et al. [24] found that the target-controlled infusion of propofol was carried out after moderate ANH, and the ANH group showed a temporarily decreased BIS value compared with that of the control group. In the study design, autologous blood was transfused immediately up to 1 h after surgery and changes in plasma concentration were observed. The results showed that there was no significant change in propofol concentration, and the BIS value increased slowly in the ANH group. BIS values at 50 and 60 min after autologous blood transfusion in the ANH group were significantly lower than those in the control group, while the increases at the other time points were less. The possible causes are analyzed as follows: The protein binding rate of propofol was up to 97% [25]. After autologous blood was transfused into vessels, the volume of blood increased and its protein binding ratio decreased, thus leading to more free propofol [24]. The free drugs entered the peripheral tissues through vascular endothelial cells, resulting in increased distribution volume. Massive free drugs distributed in the peripheral compartment led to increased distribution volume of propofol and prolonged elimination half-life. The albumin concentration in autologous blood collection before surgery was higher than the albumin concentration in plasma. With the transfusion of autologous blood, blood components changed, intravascular albumin concentration gradually increased, and colloid pressure rose. Thus, fluids in the peripheral compartment entered plasma, and broke the balance between free drugs in plasma and the peripheral compartment. The concentration of free drugs in the plasma decreased compared with the concentration in the peripheral compartment. Free drugs in the peripheral compartment rapidly returned to the plasma due to differential concentration. In addition, the collected autologous blood contained a certain concentration of the drug, and its transfusion increased the drug concentration of plasma to some extent. The above integrated factors contributed to an increase in the total amount of the drugs in plasma and the concentration of free drugs. In our study, the concentration of propofol in the two groups decreased due to hemodilution during autologous blood transfusion immediately after surgery, while there were no statistically significant differences in the concentration between the two groups at the other time points. The increase of volume after hemodilution led to the decrease in the drug concentration, which was offset by the increase in the total drug concentration after the transfusion of autologous blood. As a result, the differences in the changes in drug concentration between the two groups were free of statistical significance. However, the pharmacological effect was directly correlated with free drugs in blood. As a result, the procedures caused reduction in BIS and poor consciousness recovery.

The monitoring of muscle relaxation is essential in clinical anesthesia [26]. TOF is a clinically common index for monitoring muscle relaxation, which can provide good judgment for postoperative residual muscle relaxant [27]. Postoperative residual muscle relaxant is the main cause of delayed extubation [28]. Xu et al. [29] believed that moderate ANH (Hct < 28%) could change the acting time of vecuronium bromide and recovery time, which is mainly based on hemodilution leading to increased blood flow in vital organs, decreased plasma protein concentration, and changes in drug distribution volume. Pharmacokinetics suggests that drug action depends on distribution while elimination is mostly determined metabolically. The study by Dahaba et al. [30] on cisatracurium suggests that ED50, ED90, and ED95 (the drug achieved a 50%, 90%, and 95% effective dose, respectively) after moderate ANH, showed no significant differences compared with those of the control group. There were no significant differences in the time from the first dose to 25% TOF recovery, time from 25% recovery to 75% recovery, and time from 25% recovery to 80% recovery compared with those in the control group. In this study, there was no significant change in cisatracurium concentration in the ANH group. TOF values 40 min, 50 min, and 60 min after blood transfusion were significantly less than those in the control group, and the increases at the other time points were slow. The cause is analyzed as follows: Hemodilution causes increased distribution volume and prolonged elimination half-life [31]. With the transfusion of autologous blood, the plasma albumin concentration gradually increased and colloid osmotic pressure rose, thus more free drugs in the peripheral compartment returned to the plasma due to the differential concentration. The protein binding rate of cisatracurium is only 38%. As most of the drugs in autologous blood are free, the concentration of free drugs in plasma increased after the transfusion of autologous blood, leading to an increased proportion of free cisatracurium and drug effect. Cisatracurium is mainly metabolized by Hofmann and blood esterase. The main factors influencing Hofmann metabolism are body temperature and pH value. Studies have shown that the reduction of pH value can increase the retention time of non-depolarizing muscle relaxants and prolong the recovery time of muscle relaxation [32]. As for blood gas analysis in this study, the pH value at the end of autologous blood transfusion in the ANH group was lower than that in the control group, and Lac content was higher than that in the control group. The main reason is poor muscle relaxation recovery leading to increased anaerobic metabolism, and therefore, increasing the retention time of non-depolarizing muscle relaxants. At the same time, blood esterase concentration decreases after hemodilution and metabolism reduces accordingly, thus resulting in poor muscle relaxation recovery and delayed extubation. The above-mentioned integrated factors could cause delayed muscle relaxation recovery.

One notable limitation of this study is the absence of a non-autologous blood transfusion group following ANH, which hinders the objective evaluation of drug concentration changes. The rationale behind not including such a group is the concern that prolonged retention of autologous blood may lead to infections, extended recovery room stays, and difficulties in patient transfer. Furthermore, during the blood collection process in ANH, the volume of blood collected from elderly patients was calculated using the formula, often results in larger quantities. Therefore, it is important to vigilantly monitor hemodynamic changes in patients, continuously assess blood gases and electrolytes, and observe alterations in the internal environment and the extent of dilution.

In summary, postoperative autologous blood transfusion after moderate ANH did not significantly change the concentrations of propofol and cisatracurium but delayed the decrease of the drug concentrations. In addition, it increased the depth of anesthesia and muscle relaxant effect to some extent and delayed the recovery of respiratory function and extubation in elderly patients who underwent orthopedic surgery. Therefore, for elderly patients undergoing moderate ANH technique, special attention should be paid to hemodynamic changes after blood collection. Delayed extubation and prolonged observation time with close monitoring should be provided to prevent the occurrence of complications for patients with poor postoperative recovery of respiratory function.

## Data Availability

The datasets used and/or analysed during the current study available from the corresponding author on reasonable request. We declared that materials described in the manuscript, including all relevant raw data, will be freely available to any scientist wishing to use them for non-commercial purposes, without breaching participant confidentiality.
